# Hands off, brain off? A meta‐analysis of neuroimaging data during active and passive driving

**DOI:** 10.1002/brb3.3272

**Published:** 2023-10-12

**Authors:** Navarro Jordan, Reynaud Emanuelle

**Affiliations:** ^1^ Laboratoire d'Etude des Mécanismes Cognitifs (EA 3082) Université de Lyon Bron Cedex, Lyon France; ^2^ Institut Universitaire de France Paris France

**Keywords:** car driving, meta‐analysis, neuroergonomics, neuroimaging, visual pathways

## Abstract

**Background:**

Car driving is more and more automated, to such an extent that driving without active steering control is becoming a reality. Although active driving requires the use of visual information to guide actions (i.e., steering the vehicle), passive driving only requires looking at the driving scene without any need to act (i.e., the human is passively driven).

**Materials & Methods:**

After a careful search of the scientific literature, 11 different studies, providing 17 contrasts, were used to run a comprehensive meta‐analysis contrasting active driving with passive driving.

**Results:**

Two brain regions were recruited more consistently for active driving compared to passive driving, the left precentral gyrus (BA3 and BA4) and the left postcentral gyrus (BA4 and BA3/40), whereas a set of brain regions was recruited more consistently in passive driving compared to active driving: the left middle frontal gyrus (BA6), the right anterior lobe and the left posterior lobe of the cerebellum, the right sub‐lobar thalamus, the right anterior prefrontal cortex (BA10), the right inferior occipital gyrus (BA17/18/19), the right inferior temporal gyrus (BA37), and the left cuneus (BA17).

**Discussion:**

From a theoretical perspective, these findings support the idea that the output requirement of the visual scanning process engaged for the same activity can trigger different cerebral pathways, associated with different cognitive processes. A dorsal stream dominance was found during active driving, whereas a ventral stream dominance was obtained during passive driving. From a practical perspective, and contrary to the dominant position in the Human Factors community, our findings support the idea that a transition from passive to active driving would remain challenging as passive and active driving engage distinct neural networks.

## Graphical abstract

Driving under passive or active steering engages distinct neural networks. The findings support the idea that the output requirement of the visual scanning process engaged for the same activity can trigger different cerebral pathways, associated with different cognitive processes. This challenges Human Factors’ current position about passive driving.

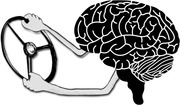

## 1 INTRODUCTION

The primary function of scanning visually our environment is to provide the information required to perform everyday tasks (Land, 2009, 2006). When considering the relationships between vision and action, mobility is key for our species, from walking on two legs to advanced technologically mediated mobility solutions. Among those technologically mediated mobility solutions, car driving is a frequent, well‐spread, and often investigated activity (Lee, 2008). Despite being so common, driving is a complex activity that has been described to engage different levels of cognitive control. Based on the combination of ergonomics knowledge (Michon, 1985, 1979) and available neuroimaging data recorded during simulated car driving, we proposed the Driver NeuroErgonomical Cascade Model (DNCM).

The DNCM describes the driving activity from the driver's intentions to vehicle trajectory through three different levels of cognitive control progressively less and less consciously controlled in a cascade fashion (Navarro et al., 2018). The most explicit level referred to as the *strategical level* (involved, e.g., in route planning and adapting the driving behavior to contextual elements such as the emergency of the driving situation, the risks associated with traffic rules violations or the driver's awareness) is engaged at first. This level of cognitive control allows drivers to set driving plans (trip goal and itinerary) and relies on three main cerebral areas: (i) the right temporoparietal junction in the right superior temporal gyrus (BA39/22) associated with attentional processes (Bzdok et al., 2013; Wilterson et al., 2021), especially in the attentional ventral network (Corbetta et al., 2008; Vossel et al., 2014), (ii) the left superior frontal gyrus (BA10) associated with the “branching” activity that consists to keep in mind relevant information for the primary task while completing another additional task (Koechlin, 2016; Koechlin et al., 1999; Koechlin & Hyafil, 2007), and (iii) the left inferior frontal gyrus (BA45) associated with semantic retrieval and working memory processes (Buckner, 1996; Gabrieli et al., 1998; Wang et al., 2019; Zhang et al., 2021).

The situation is then processed at the *tactical level* assumed to adapt driving behaviors depending on the dynamic driving environment (overtaking a car, adjusting the vehicle speed before turning, deciding to stop at a pedestrian crossing). This level of cognitive control relies on two cerebral areas: (i) the bilateral middle frontal areas, associated with complex voluntary movement planning (Cordani et al., 2022; Halsband et al., 1993), and (ii) the right middle temporal gyrus, associated with the processing of the optic flow resulting from 3D motion and visual recognition (Greenlee, 2000; Smith, Wall, et al., 2006; Wurtz, 1998).

At the third and last level of cognitive control, referred to as *operational control*, the decisions made at the strategical and tactical levels of control are implemented in terms of physical actions (such as turning the steering wheel slightly to the left and accelerating or braking heavily). According to the DNCM (Navarro et al., 2018), operational control relies on three cerebral areas: (i) the right extrastriate cortex associated with the analysis of complex visual scenes, (ii) the anterior lobe of the right cerebellum engaged in motor control (King et al., 2019; Manto et al., 2012; Schmahmann, 2019), and (iii) the right mediodorsal nucleus of the thalamus associated with the integration of various elements of complex visual scene processing (Griffiths et al., 2022; Sherman, 2007).

Although car driving could be considered an evolved form of technology‐mediated mobility, ironically even an “automobile” can be automated. There is a current trend to automate as much as possible the driving activity (Hancock, 2014; Navarro, 2019; Navarro & Hancock, 2023; Stanton & Young, 1998). Following a technocentric line, the main automation design philosophy is to replace humans whenever it is possible to do so. Driving automation is often sorted using levels of automation (LOA) (SAE International, 2018), ranging from the simplest driving assistance—LOA 0—(e.g., lane departure warning) to a completely autonomous vehicle without pedals and steering wheel (LOA 5). It is a current reality to ride highly automated driving vehicles (SAE level 3), able to control the trajectory of the car in both lateral and longitudinal dimensions, in an unknown environment that includes other vehicles, automated or not, and road users as pedestrians and cyclists, without any human action. The human behind the wheel then becomes a “passive driver” but is still required to monitor the driving environment and to regain manual control of the vehicle in case automation cannot (e.g., missing lane marking, foggy conditions, or sensor failure). It is thus an important practical issue to understand and facilitate the transition of control between passive and active driving.

To pursue this issue, drivers’ eye movements have been used as an indicator of the cognitive processes engaged while driving (Lappi & Mole, 2018), including when driving with automation (Mars & Navarro, 2012; Navarro et al., 2020, 2021, 2019) or even during the transition of control from passive to active driving (see Deniel & Navarro, 2023 for a review). Although several neuroimaging studies have been carried out on real and simulated driving, under a variety of experimental conditions (see Ware et al., 2020 for a review), only sparse information about the brain activity engaged during passive driving compared to active driving is available (Sakihara et al., 2014).

Beyond the car driving domain, an important neuroscientific finding relates to the existence of two anatomically distinct visual pathways in the cortex of mammals and humans. The first functional conception of these dual visual streams was that the ventral occipitotemporal stream was devoted to the recognition of objects and referred to as the “what” pathway, and the other dorsal occipitoparietal stream was subserving the spatial properties of objects and referred to as the “where” pathway (Ingle, 1973; Mishkin & Ungerleider, 1982; Schneider, 1969). This initial conception was refined first based on neuropsychological observations made with patient DF (Goodale et al., 1991). Goodale and Milner (1992) proposed a fresh look at the functions processed by the two streams and shifted the emphasis from the visual characteristics of the object to the goal behavior guiding the vision (Goodale & Westwood, 2004). This revised model states that the functional segregation of the streams is based on the requirements of the situation, the dorsal stream being involved in the visuospatial control of actions (e.g., grasping and reaching targets), not specifically in spatial perception (Jeannerod, 2011). The ventral stream would be used for “vision to perception” (i.e., “what”) and the dorsal stream for “vision to action” (i.e., “how”). As these seminal contributions, numerous studies and investigations have been conducted, confirming the existence and functions of the two visual pathways, and discussions about their level of relative independence or the unicity of representations inside pathways arrayed (Milner & Goodale, 2008). In brief, the ventral “what” pathway projects from the striate cortex to the inferior temporal lobe (posterior inferotemporal, central inferotemporal, and anterior inferotemporal) via occipital areas V2 and V4. The dorsal “how” pathway projects from the striate cortex, V5/MT, through the intraparietal sulcus and terminates in the superior parietal lobe.

Although active driving requires the use of visual information to guide actions (i.e., steering the vehicle), passive driving only requires looking at the driving scene without any need to act (i.e., the human passively driven is out of the action control loop). Consequently, considering driving in the light of the dual‐visual pathways for perception or action could be highly informative on the differences between the neurocognitive processes associated with these two driving modes. Indeed, it is hypothesized that the same visual input would be processed either more on the ventral stream or more on the dorsal stream depending on the output requirements: In the case of active driving, where steering is required, drivers are expected to recruit mainly the dorsal stream to perform the required goal‐oriented actions. On the contrary, in the case of passive driving, drivers are expected to engage more with the ventral stream to process their environment without the intention to act. Of course, the distinction between the two visual pathways is not expected to be as clear‐cut as described by textbooks or by neuropsychological evidence (Goodale & Milner, 1992). Here, the two pathways can be engaged in parallel by drivers, especially in the case of passive driving where drivers can supervise the drive and possibly ghost the actions required to do so.

## 2 METHODS

### 2.1 Selection of studies

**FIGURE 1:**
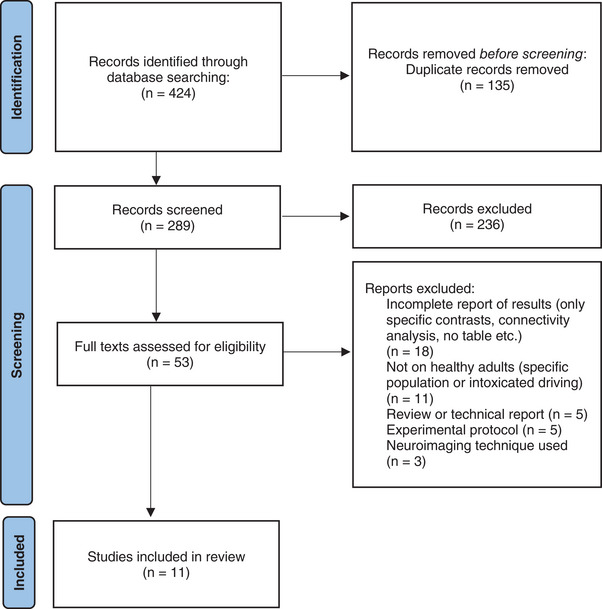
Preferred Reporting Items for Systematic Reviews and Meta‐Analysis (PRISMA) flow diagram used for the literature search.

A search in PubMed, PsycINFO, ScienceDirect, and Web of Science databases complemented with a search in Google Scholar carried out on the logical conjunction of keywords (“brain mapping” OR “functional magnetic resonance imaging” OR “fMRI” OR “positron emission tomography” OR “PET”) AND ((“driving” OR “drive”) AND (“car” OR “automobile”)) and published in English returned 424 scientific contributions at the date of September 15, 2023. By using this combination of search terms, we ensured that all published articles in English that examined the subject of the neural correlates of car driving were included in our results. The results of the search were then screened by two reviewers independently (JN and ER). In the case of disagreement, a consensus discussion solved the conflict. The screening procedure was first carried out on abstracts, and then, full texts of the remaining research articles were assessed for eligibility. The selection procedure followed the Preferred Reporting Items for Systematic Reviews and Meta‐Analysis (PRISMA) guidelines (Page et al., 2021), and the corresponding PRISMA flow diagram for eligibility of articles is presented in Figure 1. The selection was guided by a list of exclusion criteria gathering theoretical, methodological, or analytical pitfalls, namely:

1. Not on the topic of car driving,
2. No actual driving task in the experimental protocol (e.g., visuospatial task),
3. No brain data collection performed with a hemodynamic neuroimaging method,
4. No research experiment performed (e.g., theoretical, technical, or review articles),
5. Not on healthy adults (e.g., mild cognitive impairment population and elderly),
6. Study on driving while intoxicated (e.g., alcohol or antihistamines),
7. No report of foci or activation peak coordinates,
8. No neuroimaging results on whole‐brain scanning were available (e.g., results only on regions of interest),
9. Only results from conjunction or subtraction between two driving conditions were available (e.g., contrasts between two driving tasks),
10. Analyses not conducted with the general linear modeling analysis—or equivalent—framework (e.g., independent component analysis or multivariate pattern analyses).

Based on these criteria, 11 studies and 17 contrasts providing data on 187 healthy participants were ultimately included in the meta‐analysis (see Table S1 for the details of the studies included). Together, these studies comprised 285 peaks of activation.

### 2.2 Classification of the studies included

All the neuroimaging data available were grouped into two exclusive categories of simulated driving: (i) *active driving* (i.e., a situation where participants had to control both the vehicle position and its speed) and (ii) *passive driving* (i.e., a situation where participants were passively driven by a vehicle without any control on the vehicle position or its speed). A comprehensive meta‐analysis including all available functional neuroimaging results was carried out (Eickhoff et al., 2012, 2009).

### 2.3 Data analysis

A coordinate‐based meta‐analysis with an activation likelihood estimation (ALE) technique (Chein et al., 2002; Turkeltaub et al., 2002) was used to identify the anatomical locations consistently observed across neuroimaging studies (GingerALE 2.3 software (http://www.brainmap.org/ale/)).

The underlying principles of the ALE method are as follows: Based on the coordinates of activation peaks in each study selected for inclusion, the ALE method estimates the probability that an activation focus truly exists within a given voxel, with Gaussian assumptions on spatial uncertainty. An ALE map is then created as the union of probabilities over all activation foci. Clusters above a significance threshold reveal convergence across included imaging studies at the location.

The computations behind the ALE method can be roughly described as follows: To perform this meta‐analysis, coordinates of every significant activation peak for each included condition were collected, either originally in the MNI reference space or transformed from the Talairach space. For each study and at each voxel, the reported foci are considered to be the centers of 3D Gaussian probability density functions. Full widths at half maximum (FWHM) of 3D Gaussian functions depend on the sample size. Larger samples lead to smaller FWHM, reducing the uncertainty attached to the related foci, and vice versa. The probability distributions of all foci in an experiment are combined in a modeled activation (MA) map. The union of all MA maps for all the experiments included allows computing an ALE score on a voxel‐by‐voxel basis, quantifying the likelihood of convergent activations at each voxel across all included studies. Significance tests allow to threshold the union of ALE maps based on nonparametric *p*‐values with a false discovery rate (FDR) of *p* < .05. For specific contrasts between two conditions (subtraction analysis), the ALE maps are compared by subtracting one image from the other, taking into account the different sample sizes via the individual MA maps pooled to form the ALE map for an experimental condition. At the contrast level, ALE individual maps were thresholded at a level of *p* < .05 (FDR corrected) as was the pooled map for both conditions. The results were reported with a *p*‐value threshold set to *p* < .05 uncorrected, following the recommendations issued by the ALE experts against the use of FDR corrections on contrast images for small sample sizes and favoring the use of uncorrected thresholds for contrast analyses, and minimum cluster sizes set to 60 mm^3^ (Laird et al., 2005; Turkeltaub et al., 2012).

Significant clusters were overlaid onto an ICBM152 brain template in MNI space provided by the open‐source Surf Ice software (https://github.com/neurolabusc/surf‐ice).

## 3 RESULTS

### 3.1 Active driving > passive driving

**FIGURE 2:**
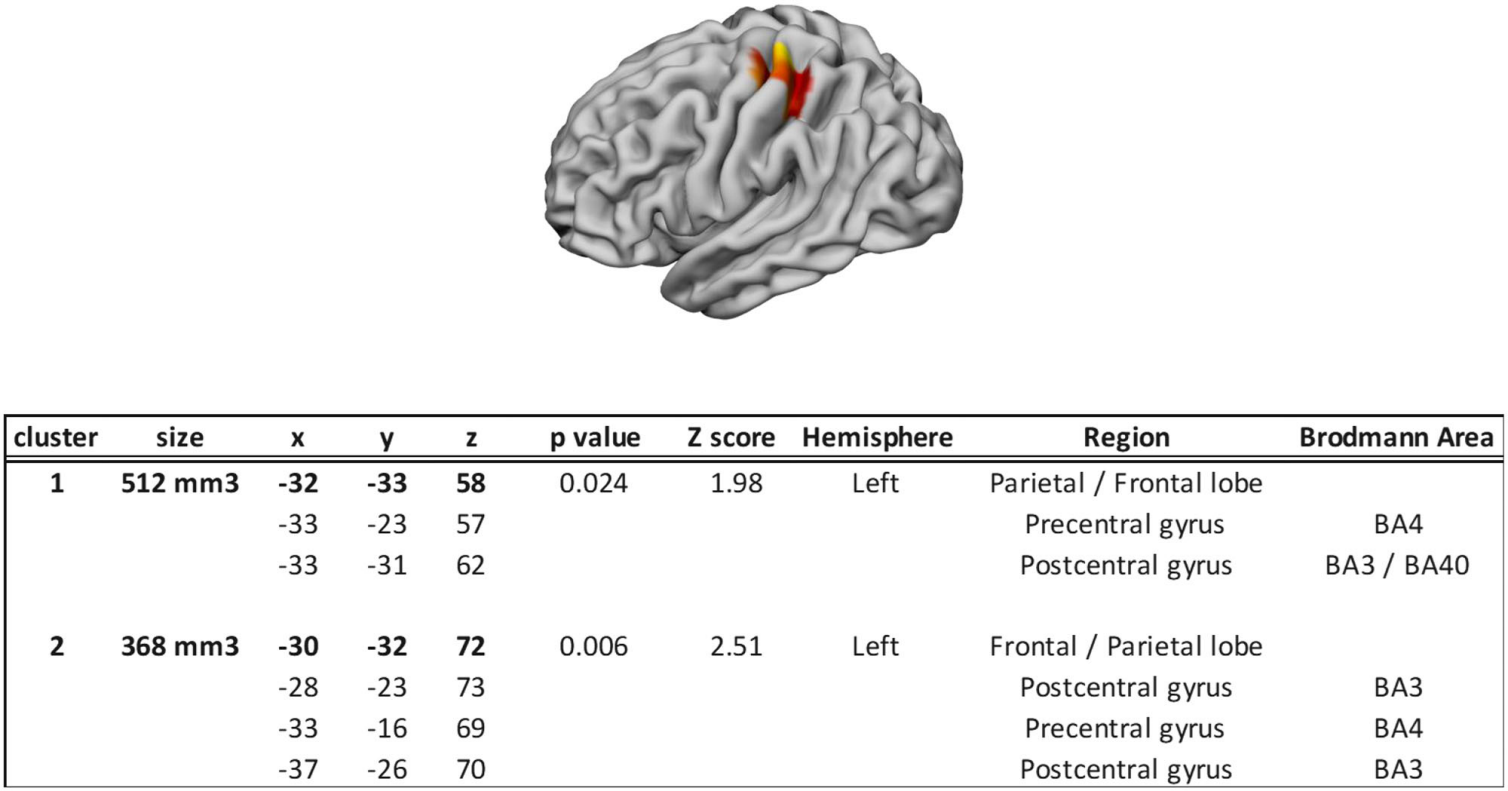
Clusters activated more consistently in active driving compared to passive driving. Clusters for the active > passive contrast. The centers of the clusters are in bold, and the peaks inside clusters are below. Coordinates are in the MNI space.

The results of the active versus passive driving contrast are given in Figure 2. They show that two brain clusters were recruited more consistently for active driving compared to passive driving, namely, the left precentral gyrus (BA3 and BA4) and the left postcentral gyrus (BA 4 and BA3/40).

### 3.2 Passive driving > active driving

**FIGURE 3:**
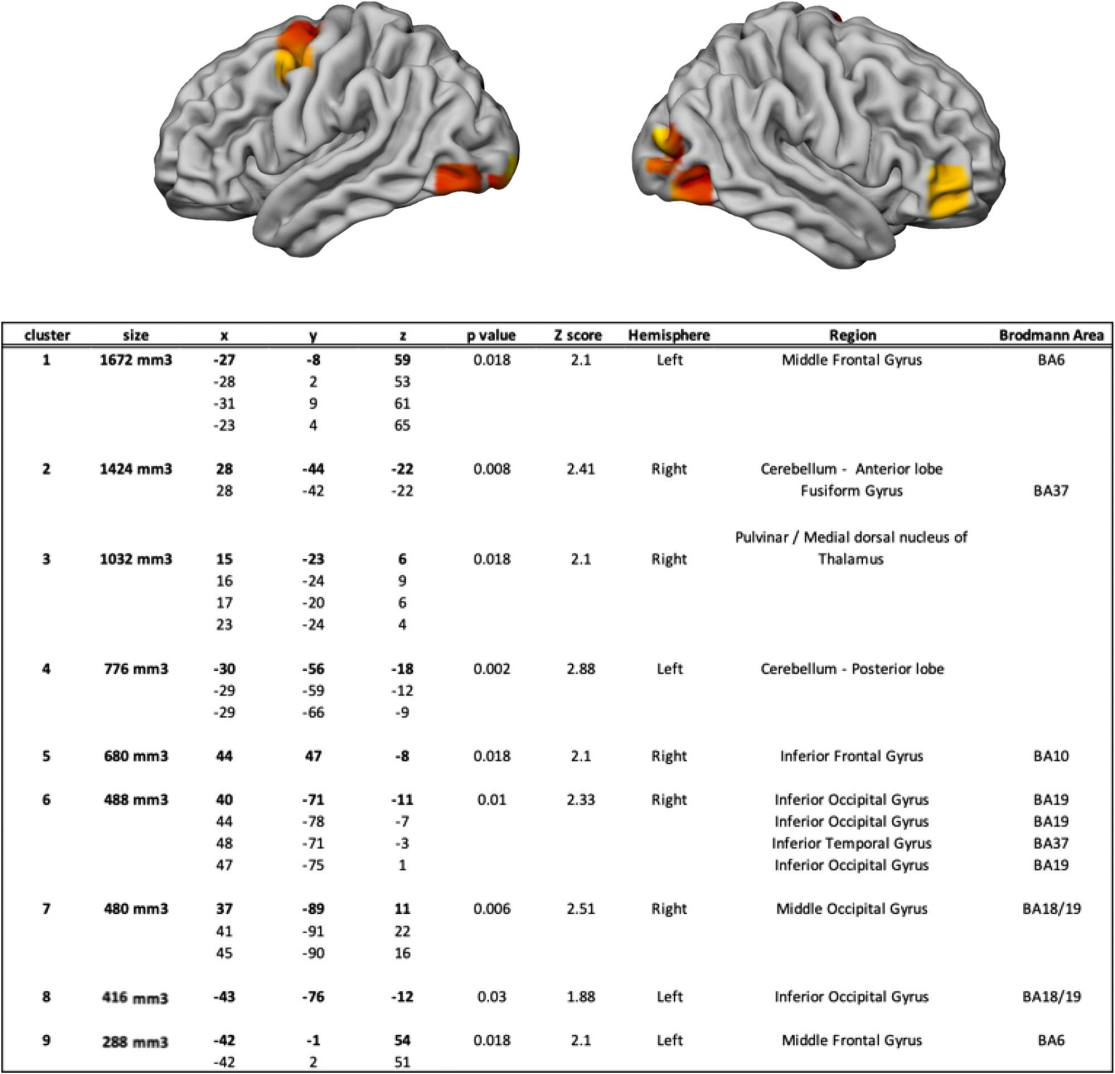
Clusters activated more consistently in passive driving compared to active driving. Clusters for the active > passive contrast. The centers of the clusters are in bold, and peaks inside clusters are below. Coordinates are in the MNI space.

The results of the passive versus active driving contrast are given in Figure 3. They show that clusters in a set of brain regions were recruited more consistently in passive driving compared to active driving, namely, the left middle frontal gyrus (BA6), the right anterior lobe and the left posterior lobe of the cerebellum, the right sub‐lobar thalamus, the right anterior prefrontal cortex (BA10), the right inferior occipital gyrus (BA17/18/19), the right inferior temporal gyrus (BA37), and the left cuneus (BA17).

## 4 DISCUSSION

### 4.1 Active driving and the dorsal pathway

The consistent activations recorded in the left *primary somatosensory cortex* (BA3) during active driving compared with passive driving could be associated with the bodily sensations processing as well as with coordinated movements. In more detail, these regions have been repeatedly observed to be engaged by ipsilateral hand–foot combination of coordinated movements (Debaere et al., 2001; Nakagawa et al., 2016; Rocca et al., 2007) and in haptic perception (Peltier et al., 2007). In our case, this consistent area of activation could be associated with the grip of the steering wheel with the right hand and the touch of the gas and/or brake pedal with the right foot. Another notable activation in active driving compared to passive driving was observed in a nearby region (BA40), an area known to be associated with motor control from basic hand and finger motor tasks (Li et al., 2020; Smith, Chen, et al., 2006), to performing everyday motor tasks, such as dressing (Wittenberg et al., 2014), through grasping (Randerath et al., 2010; Ward & Frackowiak, 2003), and elbow movements (Estévez et al., 2014; Van Dokkum et al., 2017).

The left *primary motor cortex* (BA4) was also found to be activated more consistently in active driving than in passive driving. This area is required to execute voluntary movements (Cordani et al., 2022; Halsband et al., 1993). More precisely, the area activated was previously observed as part of the network engaged for movement control through the classic neuroscientific finger tapping tasks (Anwar et al., 2016; Witt et al., 2008), but also during elbow movements (Estévez et al., 2014). This region was also reported to be part of the common car‐driving circuit to allow driving at the motor execution (“operational” level; Navarro et al., 2018).

Active driving showed more consistently activated brain areas close to the left central gyrus in both anterior and posterior locations. Other brain regions typically associated with the dorsal stream could have been expected, especially the superior parietal lobule. An explanation could be that even in passive driving condition, drivers behaved as if they were driving. Participants could have used motor imagery (Jeannerod, 1994), especially by imagining moving the steering wheel, an activity known to engage most areas associated with actual sensorimotor control (Ehrsson et al., 2003; Savaki & Raos, 2019). In some of the experiments included in the meta‐analysis, they were even instructed to do so. The absence of brain areas in the sensorimotor control network (i.e., the supplementary motor area, the cingulate motor areas, the premotor cortex, and the posterior parietal cortex) might then be attributed to drivers preparing for steering action even in the passive driving condition. This hypothesis would also be consistent with the observation of increased brain activations in brain areas corresponding to action planning and action realization observed here.

### 4.2 Passive driving and the ventral pathway

Several brain areas were activated more consistently during passive driving than during active driving. Those areas and associated functions related to passive driving requirements are discussed in the following lines.

The *left middle frontal gyrus* (BA6) was found to be activated more consistently during passive driving. In the literature, this brain region has been associated with action observation, such as reaching or grasping at rest (Molinari et al., 2013), gesture recognition (Villarreal et al., 2008), motor imagery (Wei & Luo, 2010), and anticipatory behaviors (Bianco et al., 2021; Simó et al., 2005), especially to anticipate objects’ location (Schubotz & von Cramon, 2001; Smith, 2016). During passive driving, this region would thus be involved in anticipating the vehicle's future location in space based on the observation of current motion. This interpretation is reinforced as this brain region is also engaged for spatial judgments (Kukolja et al., 2006), including during real‐world navigation in simulated driving (Spiers & Maguire, 2006).

The *cerebellum* (right anterior lobe and left posterior lobe), a structure associated with motor coordination (Ito, 1984; King et al., 2019; Manto et al., 2012) and motor error correction (Miall et al., 1993; Popa et al., 2016), was associated with passive driving. Here, in line with previous observations, the cerebellum would contribute to support the visual perception of motion (Jokisch et al., 2005; Paradis et al., 2000) and monitor visual search (Vallesi, 2014), including establishing a directional expectation for movement (Hull, 2020; Shulman et al., 1999) in the driving environment.

*The right sub‐lobar part of the thalamus* was also part of the passive driving network. Classically considered a relay for sensorimotor information to the cortex, the thalamus has numerous connections with cortical and subcortical areas (Ro et al., 2007; Sherman & Guillery, 2011). Here, the thalamus could support passive drivers’ alertness (Yanaka et al., 2010).

The right anterior prefrontal cortex (BA10), and the specific region activated here, is part of the mentalizing network (Lee, 2015), known to support the ability to assess one's mental states and to infer the mental states of others (Hein & Singer, 2008; Kalbe et al., 2010; Thornton et al., 2019). A possible interpretation of these brain activities would be that while performing passive driving individuals could evaluate their own current activity and mental state. Additionally, this region is associated with the perception of human as well as humanoid robot actions (Saygin et al., 2012). It could thus be hypothesized that during passive driving people engage in the supervision of the artificial agent (i.e., driving automation) actions. This region has also been specifically associated with stimulus‐driven attentional shifts between objects and locations (Stoppel et al., 2013). In passive driving, the visual flow of the dynamic driving scene would thus require passive drivers to switch between different elements of the environment, located at different places. Altogether, this specific region could be described as supporting the supervision of the agent actively controlling the vehicle.

The right inferior occipital gyrus (BA17/18/19) and the left cuneus (BA17), brain areas implied in visual selective attention (Kastner et al., 1998; Moran & Desimone, 1985) and visual information processing and especially perceptual identification of objects (Goodale & Milner, 1992; Grill‐Spector et al., 2001; Zeki, 1993) in its ventral part. These areas in the visual cortex have been associated with visual (Kriegeskorte et al., 2003) and audiovisual object recognition (Plank et al., 2012), 3D shape recognition (Georgieva et al., 2008) and visual object processing in general (e.g., Rose et al., 2005). The area MT/V5 is activated, as previously reported in the common car driving network (Navarro et al., 2018), to support motion perception and tracking (Culham et al., 1998; Tootell et al., 1995; Watson et al., 1993; Zeki et al., 1991). Right MT/V5 satellites are also systematically engaged during action observation including locomotion (Abdollahi et al., 2013). Here if steering action cannot be directly observed, the outcome of another agent's actions is available to participants. The numerous activations recorded in the extrastriate cortex are consistent with the need for participants to explore visually the driving scene.

The right inferior temporal gyrus (BA37) is a region of the ventral pathway (Deng et al., 2016), located very close to the lateral occipital complex implicated in object perception and recognition (Grill‐Spector et al., 2000; Malach et al., 1995), a region also engaged during action observation and execution (Abdollahi et al., 2013; Brihmat et al., 2018), the processing of objects movement (Zacks et al., 2006) with a specialization for biological (Beauchamp et al., 2002) or biologically compatible movement processing (Crescentini et al., 2011). During passive driving, this region could be engaged to (i) analyze the vehicle trajectory, resulting from human‐initiated or human‐compatible actions on steering and speed control and (ii) participate in the identification of the various objects available on the driving scene.

### 4.3 General discussion

In the studies included in the meta‐analysis, healthy participants were invited to perform a complex, skilled, and well‐known everyday task: driving. The data collected showed consistently distinct brain activations whether that activity was performed actively or passively, thus providing insight into how the vision for perception and vision for action pathways are relevant to describe real‐life behaviors.

From a theoretical perspective, the findings are in line with the idea that the output requirement of visual scanning engaged for the same activity can trigger different neural processes. Dorsal stream dominance was found during active driving, whereas ventral stream dominance was obtained during passive driving. Interestingly participants could have processed visual information during passive driving as if driving actively, after all, participants were stuck in a driving cockpit with nothing else to do but visually scan the driving scene. This was not the case. This suggests that the vision for action pathway is automatically switched on when actual action performance is required and switched off when no action performance is needed. Even if the two visual pathways can communicate (Ayzenberg et al., 2023; Kriegeskorte et al., 2003), a switching cost may apply at least from ventral dominance to dorsal dominance. This would explain the difficulties observed to regain active steering after some time spent in passive steering (Deniel & Navarro, 2023; Navarro et al., 2016; Zhang et al., 2019).

From a practical perspective, our findings support the rationale that a transition from passive to active driving (required at SAE level 3) would remain challenging no matter the quality of the monitoring engaged during the passive driving task. This assertion is at odds with the well‐established and widely accepted idea that physical control of the vehicle (i.e., steering control) and monitoring (i.e., supervision of the vehicle trajectory) of the current driving situation are two independent concepts (Merat et al., 2019). Indeed, the Trilateral Human Factors Working Group proposed that “in a situation where physical vehicle control is taken over by an automated system, the driver may still be regarded as being on the loop if (s)he is still engaged in situation monitoring” (Merat et al., 2019). The current findings suggest the reverse. Delegating physical control of the vehicle to an automated system (i.e., passive driving) de facto implies situation monitoring different from the one engaged in active driving. The visual processing of the situation goes hand in hand with action control. If no action is required, situation monitoring would be carried out differently whatever the level of implication of the drivers during passive driving. This sheds new light on the Human Factors definition and understanding of cognitive control in passive driving. One cannot expect that the drivers who are regaining active control immediately after a period of passive driving will perform as well as in active driving as the process of visual information follows different paths in the brain.

The reported findings outline that under passive driving, people's neural activations are different than under active driving. This observation was made based on several datasets where different methodologies have been used and different instructions given to participants. Still, across studies, the meta‐analysis reported that people consistently exhibit a different neural network in passive driving compared to the one deployed in active driving. This aligns with the idea that no matter the specific driving situations, conditions, or instructions, the neural regions engaged in passive driving differ from those engaged in active driving. This neuroscientific evidence speaks against the dichotomy made in Human Factors between physical control of the vehicle on the one hand and monitoring of the current driving situation on the other hand (Merat et al., 2019). From the neuroergonomics perspective adopted here, physical control of the vehicle and monitoring of the driving situation are fundamentally linked. Thus, it is impossible to delegate operational control to automation without impacting the tactical and strategical levels of control (Navarro et al., 2018). This new insight suggests that the DNCM needs to be revised. Indeed, the model described behavioral and neural activities from the driver's intentions to vehicle trajectory through three different levels of cognitive control progressively less and less consciously controlled in a cascade fashion (Navarro et al., 2018). The reported findings indicate that the cascade nature of the model is unsatisfying and that feedback from the operational level to the tactical and strategical levels should be added. It can be hypothesized that passive and active steering operations act as a bottom–up process able to counterbalance the cascade top–down process described in the model.

### 4.4 Limitations

Quantitative meta‐analyses are often considered the highest level of scientific proof thanks to the combination of datasets gained through several experiments. This combination not only offers the possibility to add the observations made on different participants but also to consider the common cerebral changes recorded across experiments despite different experimental setups and other various methodological choices. However, no control is possible on the experimental manipulations made for the data included in the meta‐analysis. Here, the driving activity was systematically engaging a vehicle moving in an environment, the vehicle trajectory being controlled either actively or passively. But the driving activity may include a variety of subtasks that are not strictly equivalent (McKnight & Adams, 1970) and cognitively controlled at different levels (Navarro et al., 2018). It is thus possible that the reported findings did not cover the complete range of neural differences between passive and active driving. Rather, the reported findings should be considered the minimum differences between passive and active driving. Further investigations are required to investigate in more detail the differences between passive and active driving, possibly depending on the specific driving situations (e.g., steering along a bendy road, interacting with other drivers at an intersection, and navigating). Still, the reported minimal differences between passive and active driving constitute a solid basis observable consistently across 11 experiments with various original protocols and unstandardized driving activity.

In the same vein, in all passive driving studies, participants were expected to monitor the vehicle moves in the driving environment whereas active driving consisted of a variety of different actions to be performed (e.g., negotiating an intersection, navigating in an unknown environment, and follow a given route). The larger passive driving network relative to the active driving network could be attributed to a more homogenous passive driving situation across studies. Indeed, monitoring different driving actions might be more similar than performing different actions. Moreover, dorsal pathway activations may be task‐specific and could also be subdivided into distinct neural pathways (Rizzolatti & Matelli, 2003). To specifically address the question of the transition of control between passive and active driving (i.e., takeover), such transitions should be investigated directly. Neural activations before, during, and after transitions of control would provide insightful data not only about the transition of control during driving but also shed some light on the processes engaged while switching from ventral to dorsal pathways. Another possibility to refine our understanding of the processes engaged in passive driving is to manipulate the quality of the human supervision of passive driving (no supervision required, or tight supervision required with a regular return to active driving, for instance).

More broadly, meta‐analyses also suffer from possible publication and selection biases. Indeed, only published articles providing raw data can be included in quantitative meta‐analyses. There is thus a possibility that unpublished and/or unavailable data would impact the reported findings.

Finally, the question of ecological validity (i.e., transferability of the experimental findings to real‐life driving) can be raised as most data were collected under simulated driving conditions with physical constraints due to the neuroimaging recording device. More real‐life investigations would be required to confirm the validity of the reported findings in real‐life conditions.

## 5 CONCLUSION

The meta‐analysis reported here aimed at addressing the question of the cerebral bases of passive and active driving. The results showed that active driving engages more consistently areas of the dorsal pathway compared to passive driving, as an action needs to be performed. On the contrary, the passive driving mode engages a more ventral set of cerebral areas, probably associated with the visual analysis of the driving scene and the supervision of the automation in charge of driving. It should be very clear that these two dorsal and ventral pathways of activation supporting driving are not exclusive from one another, but the switching cost between these two pathways could contribute to explaining the difficulties faced by humans when a transition from passive driving to active driving is required. The same visual information of the driving environment is processed differently in passive driving than in active driving. One cannot expect that the drivers who are regaining active control immediately after a period of passive driving will perform as if the same processes were engaged. Therefore, these results should be incorporated into future research and development on automated driving.

## AUTHOR CONTRIBUTIONS

Navarro Jordan and Reynaud Emanuelle contributed to the conception, the analyses, and the writing of the article.

## CONFLICT OF INTEREST STATEMENT

The authors have no conflicts of interest to disclose.

### PEER REVIEW

The peer review history for this article is available at https://publons.com/publon/10.1002/brb3.3272.

## Supporting information

Supporting information

## Data Availability

The data that support the findings of this study are available from the authors upon request.
