# Supplementary material for: Hands off, brain off? A meta‐analysis of neuroimaging data during active and passive driving
Source: Brain Behav. 2023 Oct 12;13(12):e3272. doi: 10.1002/brb3.3272 (PMC10726911; doi:10.1002/brb3.3272)
Supplement: Supplementary file 1 — Supporting information [file BRB3-13-e3272-s001.docx]

**Are autonomous cars’ passengers still driving?
A meta-analysis of neuroimaging data with and without active steering control**

Navarro Jordan^1,2^ & Reynaud Emanuelle^1^

^1^Laboratoire d’Etude des Mécanismes Cognitifs (EA 3082), Université de Lyon, France

^2^Institut Universitaire de France, Paris, France

Laboratoire d’Etude des Mécanismes Cognitifs, Université de Lyon, 5, avenue Pierre Mendès-France, 69676 Bron Cedex, France

Phone: +33 4 78 77 24 31

Email: jordan.navarro@univ-lyon2.fr

| Table 1 |  |  |  |  |  |  |
| --- | --- | --- | --- | --- | --- | --- |
| Overview of the studies included in the meta-analysis. | | | |  |  |  |
|  |  |  |  |  |  |  |
| 1^st^ Author | Year | Journal | Original contrast in the given study | Table number | N | Driving category |
| Mader | 2009 | Neuroscience Letters | Unfamiliar route | Table 1 | 16 | Passive |
|  |  |  | Familiar route | Table 1 | 16 | Passive |
| Jeong | 2006 | Annals of Nuclear Medecine | Active driving > control | Table 1 | 10 | Active |
|  |  |  | Passive driving > control | Table 1 | 10 | Passive |
| Schweizer | 2013 | Frontiers in Human Neuroscience | Right and left turns | Table 1 | 16 | Active |
|  |  |  | Straight driving | Table 1 | 16 | Active |
|  |  |  | Left turns +traffic and left turns + traffic + audio | Table 1 | 16 | Active |
| Graydon | 2004 | Transportation Research Part F | Visual detection > fixation baseline | Table 2 | 6 | Passive |
| Horikawa | 2005 | Brain and Cognition | Driving > rest | Table 1 | 15 | Active |
|  |  |  | Passive > rest | Table 1 | 15 | Passive |
| Kan | 2013 | Medical Physics | Driving > fixation | Table 4 | 16 | Active |
| Walter | 2001 | Neuroreport | Driving > visual scene | Table 1 | 12 | Active |
|  |  |  | Passive driving > visual scene | Table 1 | 12 | Passive |
| Uchiyama | 2012 | Transportation Research Part F | Driving > rest | Table 1 | 18 | Active |
| Choi | 2017 | ﻿Journal of Physiological Anthropology | Driving only > control | Table 2 | 15 | Active |
| Sakai | 2018 | Scientific Reports | Passive driving > control | Supp. Mat. | 34 | Passive |
| Just | 2008 | Brain Research | Driving alone > fixation baseline | Section 2.2 | 29 | Active |

REFERENCES OF THE STUDIES INCLUDED IN THE META-ANALYSIS

Choi, M.H., Kim, H.S., Yoon, H.J., Lee, J.C., Baek, J.H., Choi, J.S., Tack, G.R., Min, B.C., Lim, D.W., Chung, S.C., 2017. Increase in brain activation due to sub-tasks during driving: fMRI study using new MR-compatible driving simulator. J. Physiol. Anthropol. 36, 11. doi:10.1186/s40101-017-0128-8

Graydon, F.X., Young, R.A., Benton, M.D., Genik, R.J., Posse, S., Hsieh, L., Green, C., 2004. Visual event detection during simulated driving: Identifying the neural correlates with functional neuroimaging. Transp. Res. Part F Traffic Psychol. Behav. 7, 271–286. doi:10.1016/j.trf.2004.09.006

Horikawa, E., Okamura, N., Tashiro, M., Sakurada, Y., Maruyama, M., Arai, H., Yamaguchi, K., Sasaki, H., Yanai, K., Itoh, M., 2005. The neural correlates of driving performance identified using positron emission tomography. Brain Cogn. 58, 166–171. doi:10.1016/j.bandc.2004.10.002

Jeong, M.J., Tashiro, M.T., Singh, L.N.S., Yamaguchi, K.Y., 2006. Functional brain mapping of actual car-driving using [ 18 F ] FDG-PET. Ann. Nucl. Med. 20, 623–628.

Just, M.A., Keller, T.A., Cynkar, J., 2008. A decrease in brain activation associated with driving when listening to someone speak. Brain Res. 1205, 70–80. doi:10.1016/j.brainres.2007.12.075

Kan, K., Schweizer, T. a, Tam, F., Graham, S.J., 2013. Methodology for functional MRI of simulated driving. Med. Phys. 40, 012301. doi:10.1118/1.4769107

Mader, M., Bresges, A., Topal, R., Busse, A., Forsting, M., Gizewski, E.R., 2009. Simulated car driving in fMRI—Cerebral activation patterns driving an unfamiliar and a familiar route. Neurosci. Lett. 464, 222–227. doi:10.1016/j.neulet.2009.08.056

Sakai, H., Ando, T., Sadato, N., Uchiyama, Y., 2018. Speed-related activation in the mesolimbic dopamine system during the observation of driver-view videos. Sci. Rep. 8, 711. doi:10.1038/s41598-017-18792-y

Schweizer, T. a, Kan, K., Hung, Y., Tam, F., Naglie, G., Graham, S.J., 2013. Brain activity during driving with distraction: an immersive fMRI study. Front. Hum. Neurosci. 7, 53. doi:10.3389/fnhum.2013.00053

Uchiyama, Y., Toyoda, H., Sakai, H., Shin, D., Ebe, K., Sadato, N., 2012. Suppression of brain activity related to a car-following task with an auditory task: An fMRI study. Transp. Res. Part F Traffic Psychol. Behav. 15, 25–37. doi:10.1016/j.trf.2011.11.002

Walter, H., Vetter, S.C., Grothe, J., Wunderlich, A.P., Hahn, S., Spitzer, M., 2001. The neural correlates of driving. Neuroreport 12, 1763–1767. doi:10.1097/00001756-200106130-00049
